# Supplementary material for: Hemodynamic differences between women and men with elevated blood pressure in China: A non-invasive assessment of 45,082 adults using impedance cardiography
Source: PLoS One. 2022 Jun 14;17(6):e0269777. doi: 10.1371/journal.pone.0269777 (PMC9197037; doi:10.1371/journal.pone.0269777)
Supplement: S1 Fig — (PDF) [file pone.0269777.s001.pdf]

S1 Figure. Median Cardiac Output, Cardiac Index, Systemic Vascular Resistance, and Systemic Vascular Resistance Index by Age Among Women and Men with Systolic Blood Pressure ≥140 mmHg or Diastolic Blood Pressure ≥90 mmHg.

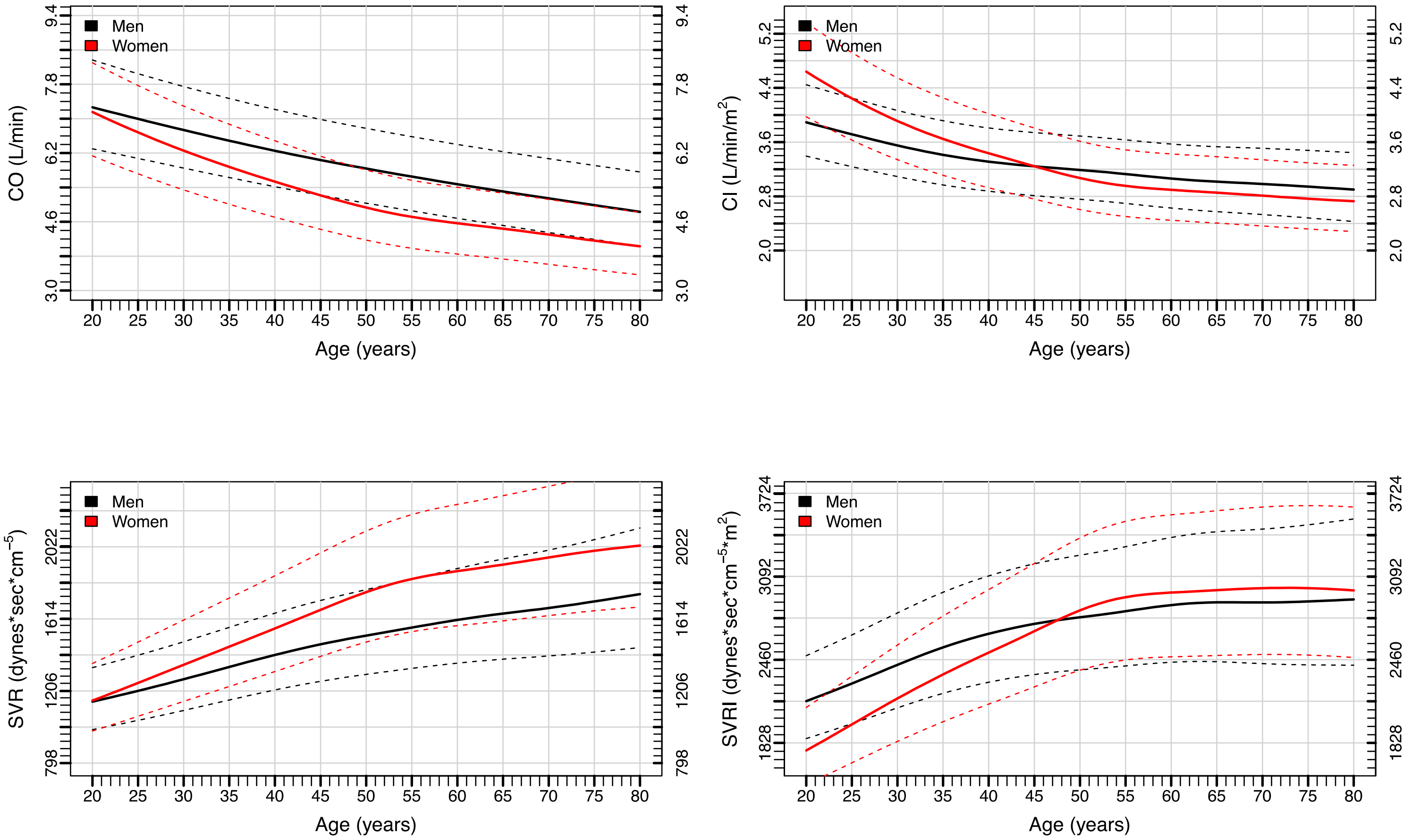

Solid lines represent the median. Dashed lines represent the 25th and 75th percentile.

Abbreviations: CO, cardiac output; CI, cardiac index; SVR, systemic vascular resistance; SVRI, systemic vascular resistance index.
