# Supplementary material for: Hemodynamic differences between women and men with elevated blood pressure in China: A non-invasive assessment of 45,082 adults using impedance cardiography
Source: PLoS One. 2022 Jun 14;17(6):e0269777. doi: 10.1371/journal.pone.0269777 (PMC9197037; doi:10.1371/journal.pone.0269777)
Supplement: S2 Fig — (PDF) [file pone.0269777.s002.pdf]

S2 Figure. Cardiac Output and Cardiac Index Density Plots Overlap Between Women and Men with Systolic Blood Pressure ≥140 mmHg or Diastolic Blood Pressure ≥90 mmHg, by Age Category.

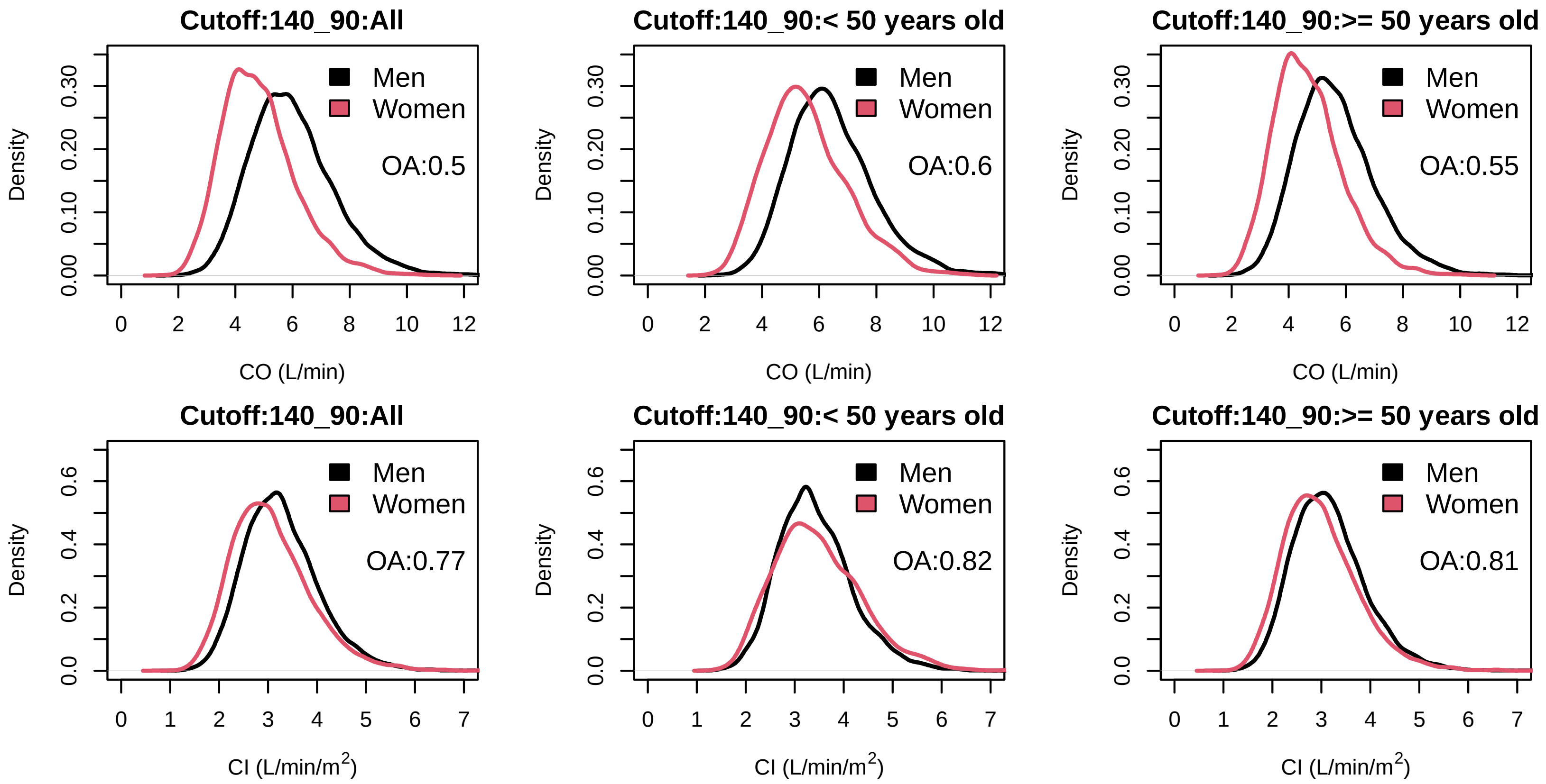

CO, cardiac output; CI, cardiac index; OA, overlapping area.
