# Supplementary material for: Hemodynamic differences between women and men with elevated blood pressure in China: A non-invasive assessment of 45,082 adults using impedance cardiography
Source: PLoS One. 2022 Jun 14;17(6):e0269777. doi: 10.1371/journal.pone.0269777 (PMC9197037; doi:10.1371/journal.pone.0269777)
Supplement: S1 Table — (PDF) [file pone.0269777.s004.pdf]

S1 Table . Sex Differences in Clinical and Hemodynamic Variables By Age Group Among Adults with Systolic Blood Pressure ≥140 mmHg or Diastolic Blood Pressure ≥90 mmHg.

	**All**			**< 50 years old**			**≥ 50 years old**		
	Women N=7593	Men N=12711	P value	Women N = 1246	Men N = 5439	P value	Women N = 6347	Men N = 7272	P value
Age (years), mean (SD)	58.13 (10.02)	51.27 (12.72)	< 0.001	42.1 (6.85)	39.22 (7.11)	< 0.001	61.27 (7.12)	60.29 (7.43)	< 0.001
BMI (kg/m2), mean (SD)	25.02 (3.51)	25.89 (3.29)	< 0.001	24.63 (3.84)	26.49 (3.51)	< 0.001	25.1 (3.44)	25.44 (3.05)	< 0.001
Obese (BMI ≥27.5 kg/m2), n(%)	1623 (21.37%)	3560 (28.01%)	< 0.001	248 (19.9%)	1884 (34.64%)	< 0.001	1375 (21.66%)	1676 (23.05%)	0.06
Region, n (%)			< 0.001			< 0.001			<0.001
East	2776 (36.56%)	5834 (45.9%)		542 (43.5%)	2653 (48.78%)		2234 (35.2%)	3181 (43.74%)	
North	1737 (22.88%)	1869 (14.7%)		219 (17.58%)	669 (12.3%)		1518 (23.92%)	1200 (16.5%)	
South	1360 (17.91%)	1792 (14.1%)		200 (16.05%)	785 (14.43%)		1160 (18.28%)	1007 (13.85%)	
Southwest	1720 (22.65%)	3216 (25.3%)		285 (22.87%)	1332 (24.49%)		1435 (22.61%)	1884 (25.91%)	
Blood pressure in mmHg, mean (SD)									
Systolic	150.75 (13.68)	147.48 (13.02)	< 0.001	144.68 (13.71)	144.48 (12.18)	0.60	151.94 (13.36)	149.72 (13.18)	< 0.001
Diastolic	86.31 (10.17)	91.43 (9.46)	< 0.001	90.5 (8.98)	92.97 (9.25)	< 0.001	85.49 (10.19)	90.28 (9.44)	< 0.001
Hypertension phenotype, n (%)									
Predominantly cardiac (High CI with low/normal SVRI)	923 (12.16%)	1957 (15.40%)	< 0.001	318 (25.52%)	1063 (19.54%)	< 0.001	263 (4.14%)	894 (12.29%)	< 0.001
Predominantly vascular (Low/normal CI with high SVRI)	5671 (74.69%)	8952 (70.43%)	< 0.001	732 (58.75%)	3474 (63.87%)	< 0.001	4939 (77.82%)	5478 (75.33%)	< 0.001
Low/normal CI & SVRI	981 (12.92%)	1737 (13.67%)	0.14	190 (15.25%)	872 (16.03%)	0.522	791 (12.46%)	865 (11.89%)	0.33
High CI & SVRI	18 (0.24%)	65 (0.51%)	0.004	6 (0.48%)	30 (0.55%)	0.928	12 (0.19%)	35 (0.48%)	0.006
ICG parameters, mean (SD)									
Heart rate (bpm)	69.69 (11.47)	70.49 (11.66)	< 0.001	73.27 (11.99)	72.47 (11.53)	0.03	68.99 (11.23)	69.01 (11.54)	0.91
Stroke volume (mL)	70.01 (17.95)	86.01 (21.19)	< 0.001	76.73 (18.25)	90.22 (21.48)	< 0.001	68.69 (17.59)	82.86 (20.42)	< 0.001
CO (L/min)	4.83 (1.29)	5.98 (1.47)	< 0.001	5.57 (1.42)	6.44 (1.46)	< 0.001	4.68 (1.21)	5.63 (1.37)	< 0.001
CI ($L/min/m^2$)	3.06 (0.8)	3.27 (0.76)	< 0.001	3.47 (0.87)	3.42 (0.75)	0.06	2.99 (0.76)	3.16 (0.75)	< 0.001
SVR ($dynes \cdot sec \cdot cm^{-5}$)	1917.42 (549.88)	1564.95 (417.38)	< 0.001	1666.98 (468.62)	1442.02 (363.25)	< 0.001	1966.59 (551.29)	1656.9 (431.37)	< 0.001

	**All**			**< 50 years old**			**≥ 50 years old**		
	Women N=7593	Men N=12711	P value	Women N = 1246	Men N = 5439	P value	Women N = 6347	Men N = 7272	P value
SVRI (dynes·sec·cm^{-5}·m^{2})	3011.81 (850.4)	2843.28 (726.62)	< 0.001	2674.73 (746.86)	2707.12 (675.2)	0.14	3077.98 (853.85)	2945.1 (746.81)	< 0.001

SD= Standard Deviation, BMI= Body Mass Index, ICG= Impedance Cardiography, SVR= Systemic Vascular Resistance, SVRI= Systemic Vascular Resistance Index, CO= Cardiac Output, CI= Cardiac Index.
