# Supplementary material for: Hemodynamic differences between women and men with elevated blood pressure in China: A non-invasive assessment of 45,082 adults using impedance cardiography
Source: PLoS One. 2022 Jun 14;17(6):e0269777. doi: 10.1371/journal.pone.0269777 (PMC9197037; doi:10.1371/journal.pone.0269777)
Supplement: S2 Table — (PDF) [file pone.0269777.s005.pdf]

S2 Table. Sex Differences in Clinical and Hemodynamic Variables by Nearest Neighbor Propensity Score Matched Subgroups.

	All 1:1 Matching			< 50 years old 1:1 Matching			≥ 50 years old 1:1 Matching		
	Women N=15,888	Men N= 15,888	P value	Women N = 4,384	Men N = 4,384	P value	Women N = 11,504	Men N = 11,504	P value
Age, years mean (SD)	54.46 (11.83)	53.45 (12.72)	<0.001	39.3 (8.07)	39.27 (7.5)	0.84	60.24 (6.9)	59.68 (7.24)	<0.001
BMI (kg/m2), mean (SD)	24.4 (3.49)	24.53 (2.95)	<0.001	23.49 (3.72)	23.65 (3.3)	0.04	24.75 (3.33)	24.97 (2.94)	<0.001
Obese (BMI ≥27.5 kg/m2), n(%)	2733 (17.2%)	2284 (14.38%)	<0.001	585 (13.34%)	483 (11.02%)	<0.001	2148 (18.67%)	2086 (18.13%)	0.30
Region, n (%)			<0.001			0.26			<0.001
East	5965 (48.74%)	6273 (51.26%)		1956 (50.46%)	1920 (49.54%)		4009 (46.58%)	4597 (53.42%)	
North	3665 (51.84%)	3405 (48.16%)		779 (47.94%)	846 (52.06%)		2886 (55.98%)	2269 (44.02%)	
South	2737 (50.39%)	2695 (49.61%)		686 (49.67%)	695 (50.33%)		2051 (52.32%)	1869 (47.68%)	
South West	3521 (50.04%)	3515 (49.96%)		963 (51.06%)	923 (48.94%)		2558 (48.02%)	2769 (51.98%)	
Blood pressure in mmHg, mean (SD)									
Systolic	139.02 (15.71)	138 (13.98)	<0.001	131.21 (13.19)	131.52 (11.06)	0.04	142 (15.57)	140.36 (14.57)	<0.001
Diastolic	82.64 (9.00)	83.62 (8.39)	<0.001	83.32 (7.85)	83.37 (8.05)	0.23	82.37 (9.38)	84.61 (8.32)	<0.001
ICG parameters, mean (SD)									
CO (L/min)	5.01 (1.35)	5.87 (1.43)	<0.001	5.75 (1.41)	6.39 (1.42)	<0.001	4.73 (1.21)	5.63 (1.35)	<0.001
CI (L/min/m2)	3.19 (0.84)	3.31 (0.78)	<0.001	3.64 (0.88)	3.59 (0.77)	0.013	3.02 (0.76)	3.18 (0.74)	<0.001
SVR (dynes·sec·cm-5)	1743.94 (523.42)	1475.6 (406.58)	<0.001	1470.91 (411.34)	1306.85 (315.19)	<0.001	1847.99 (524.09)	1552.76 (410.64)	<0.001
SVRI (dynes·sec·cm-5·m2)	2734.1 (809.92)	2606.44 (691.7)	<0.001	2325.95 (658.03)	2322.25 (556.02)	0.78	2889.64 (808.27)	2741.28 (702.93)	<0.001

SD= Standard Deviation, BMI= Body Mass Index, ICG= Impedance Cardiography, SVR= Systemic Vascular Resistance, SVRI= Systemic Vascular Resistance Index, CO= Cardiac Output, CI= Cardiac Index.
Propensity Score Generation Model: Gender ~ Age + BMI + SBP + DBP + Region
