# Supplementary material for: Hemodynamic differences between women and men with elevated blood pressure in China: A non-invasive assessment of 45,082 adults using impedance cardiography
Source: PLoS One. 2022 Jun 14;17(6):e0269777. doi: 10.1371/journal.pone.0269777 (PMC9197037; doi:10.1371/journal.pone.0269777)
Supplement: S3 Table — (PDF) [file pone.0269777.s006.pdf]

S3 Table. Unadjusted and Sequentially-Adjusted Association of Female Sex with Cardiac Output, Cardiac Index, Systemic Vascular Resistance, and Systemic Vascular Resistance Index, Overall and by Age Categories Among Adults Systolic Blood Pressure ≥140 mmHg or Diastolic Blood Pressure ≥90 mmHg, by Age Category.

**Hemodynamic Variable**	**Female Sex β Coefficient (95% CI)**		
	Unadjusted Model	Adjusted Model 1*	Adjusted Model 2**
**Cardiac Output,** (L/min)			
Overall	-1.15 (-1.19, -1.11)	-0.87 (-0.91, -0.84)	-0.87 (-0.91, -0.83)
<50 years old	-0.87 (-0.96, -0.78)	-0.71 (-0.8, -0.63)	-0.67 (-0.76, -0.58)
≥50 years old	-0.95 (-1, -0.91)	-0.91 (-0.96, -0.87)	-0.91 (-0.96, -0.87)
**Cardiac Index,** (L/min/m^2)			
Overall	-0.21 (-0.23, -0.19)	-0.09 (-0.11, -0.07)	-0.13 (-0.16, -0.11)
<50 years old	0.05 (0, 0.09)[a]	0.13 (0.08, 0.17)	0.02 (-0.03, 0.06)[b]
≥50 years old	-0.17 (-0.2, -0.15)	-0.15 (-0.18, -0.13)	-0.17 (-0.2, -0.15)
**Systemic Vascular Resistance,** (dynes·sec·cm^{-5})			
Overall	352.47 (339.07, 365.87)	273.29 (259.87, 286.72)	275.51 (262.02, 289)
<50 years old	224.95 (201.24, 248.66)	181.64 (158.55, 204.72)	183.61 (159.99, 207.23)
≥50 years old	309.69 (293.16, 326.22)	298.18 (281.67, 314.69)	299.21 (282.67, 315.74)
**Systemic Vascular Resistance Index,** (dynes·sec·cm^{-5}·m^2)			
Overall	168.52 (146.48, 190.56)	67.26 (44.85, 89.68)	112.69 (90.99, 134.39)
<50 years old	-32.39 (-74.82, 10.04)[c]	-108.78 (-150.29, -67.26)	1.48 (-39.14, 42.09)[d]
≥50 years old	132.85 (105.97, 159.74)	114.16 (87.29, 141.03)	136.87 (110.84, 162.89)

* Model 1 was adjusted for age and region
** Model 2 was adjusted for age, region, and body mass index

[a]P value=0.06
[b]P value=0.51
[c]P value=0.13
[d]P value=0.94
All other P values <0.001
