# Supplementary material for: Hemodynamic differences between women and men with elevated blood pressure in China: A non-invasive assessment of 45,082 adults using impedance cardiography
Source: PLoS One. 2022 Jun 14;17(6):e0269777. doi: 10.1371/journal.pone.0269777 (PMC9197037; doi:10.1371/journal.pone.0269777)
Supplement: Attachment — Submitted filename: PONE-D-21-16564_R1_Response_2022_02_11.docx [file pone.0269777.s007.docx]

**Response To Reviewers**

PONE-D-21-16564: Hemodynamic Differences Between Women and Men with Elevated Blood Pressure in China

We appreciate the academic editor’s and reviewers’ comments, which have helped us to improve our manuscript. We have responded to each comment (reproduced in **bold**) and detailed our changes to the manuscript. In this document, quoted text is presented indented and new additions (or relevant text when specified) underlined. The page and line numbers that we reference below are based on the clean version of our revised manuscript. New references are listed at the end of each response. We first addressed the academic editor’s comments and then each of the two reviewers.

**ACADEMIC EDITOR**

**Journal Requirements:**

**When submitting your revision, we need you to address these additional requirements.**

**1. Please ensure that your manuscript meets PLOS ONE's style requirements, including those for file naming. The PLOS ONE style templates can be found at** 
[**https://journals.plos.org/plosone/s/file?id=wjVg/PLOSOne_formatting_sample_main_body.pdf**](https://journals.plos.org/plosone/s/file?id=wjVg/PLOSOne_formatting_sample_main_body.pdf) **and** 
[**https://journals.plos.org/plosone/s/file?id=ba62/PLOSOne_formatting_sample_title_authors_affiliations.pdf**](https://journals.plos.org/plosone/s/file?id=ba62/PLOSOne_formatting_sample_title_authors_affiliations.pdf)

Response: We have reviewed the style requirements and made the necessary adjustments.

**2. Thank you for stating the following financial disclosure: "This study was self-funded."**

**At this time, please address the following queries:**

**a) Please clarify the sources of funding (financial or material support) for your study. List the grants or organizations that supported your study, including funding received from your institution. 
b) State what role the funders took in the study. If the funders had no role in your study, please state: “The funders had no role in study design, data collection and analysis, decision to publish, or preparation of the manuscript.”
c) If any authors received a salary from any of your funders, please state which authors and which funders.
d) If you did not receive any funding for this study, please state: “The authors received no specific funding for this work.”**

**Please include your amended statements within your cover letter; we will change the online submission form on your behalf.**

Response: Thank you. We modified our funding statement, as shown below (underlined text is new):

**Source of funding**:

The authors received no specific funding for this work.

**3. Thank you for stating the following in the Competing Interests section:**

**"Yuan Lu is supported by the National Heart, Lung, and Blood Institute (K12HL138037) and the Yale Center for Implementation Science. She was a recipient of a research agreement, through Yale University, from the Shenzhen Center for Health Information for work to advance intelligent disease prevention and health promotion. Erica S. Spatz receives support from the Food and Drug Administration to support projects within the Yale-Mayo Clinic Center of Excellence in Regulatory Science and Innovation (CERSI); the National Institute on Minority Health and Health Disparities (U54MD010711-01) to study precision-based approaches to diagnosing and preventing hypertension; and the National Institute of Biomedical Imaging and Bioengineering (R01EB028106-01) to study a cuff-less blood pressure device. Xin Zheng is supported by the CAMS Innovation Fund for Medical Science (2016-I2M-1-006), the National Key Research and Development Program (2016YFE0103800) from the Ministry of Science and Technology of China. Hui Lu is supported by the National Key R&D Program of China (2018YFC0910500) and Neil Shen’s SJTU Medical Research Fund. Zheng J. Ma is affiliated with Beijing Li-Heng Medical Technologies, Ltd, which designed the ICG device used in this study. Harlan Krumholz works under contract with the Centers for Medicare & Medicaid Services to support quality measurement programs; was a recipient of a research grant, through Yale, from Medtronic and the U.S. Food and Drug Administration to develop methods for post-market surveillance of medical devices; was a recipient of a research grant with Medtronic and is the recipient of a research grant from Johnson & Johnson, through Yale University, to support clinical trial data sharing; was a recipient of a research agreement, through Yale University, from the Shenzhen Center for Health Information for work to advance intelligent disease prevention and health promotion; collaborates with the National Center for Cardiovascular Diseases in Beijing; receives payment from the Arnold & Porter Law Firm for work related to the Sanofi clopidogrel litigation, from the Ben C. Martin Law Firm for work related to the Cook IVC filter litigation, and from the Siegfried and Jensen Law Firm for work related to Vioxx litigation; chairs a Cardiac Scientific Advisory Board for UnitedHealth; was a participant/participant representative of the IBM Watson Health Life Sciences Board; is a member of the Advisory Board for Element Science, the Advisory Board for Facebook, and the Physician Advisory Board for Aetna; and is the founder of HugoHealth, a personal health information platform, and co-founder of Refactor Health, an enterprise healthcare AI-augmented data management company. The other co-authors report no potential competing interests."**

**Please confirm that this does not alter your adherence to all PLOS ONE policies on sharing data and materials, by including the following statement: "This does not alter our adherence to  PLOS ONE policies on sharing data and materials.” (as detailed online in our guide for authors** [**http://journals.plos.org/plosone/s/competing-interests**](http://journals.plos.org/plosone/s/competing-interests)**).  If there are restrictions on sharing of data and/or materials, please state these. Please note that we cannot proceed with consideration of your article until this information has been declared.**

**Please include your updated Competing Interests statement in your cover letter; we will change the online submission form on your behalf.**

Response: We reviewed the guide for authors and confirm that such competing interests do not alter our adherence to PLOS ONE policies on sharing data and materials. The revised disclosure now reads as below (underlined text is new):

**DISCLOSURES**

**Yuan Lu** is supported by the National Heart, Lung, and Blood Institute (K12HL138037) and the Yale Center for Implementation Science. **Erica S. Spatz** receives support from the Food and Drug Administration to support projects within the Yale-Mayo Clinic Center of Excellence in Regulatory Science and Innovation (CERSI); the National Institute on Minority Health and Health Disparities (U54MD010711-01) to study precision-based approaches to diagnosing and preventing hypertension; and the National Institute of Biomedical Imaging and Bioengineering (R01EB028106-01) to study a cuff-less blood pressure device. **Xin Zheng** is supported by the CAMS Innovation Fund for Medical Science (2016-I2M-1-006) and the National Key Research and Development Program (2016YFE0103800) from the Ministry of Science and Technology of China. **Hui Lu** is supported by the National Key R&D Program of China (2018YFC0910500) and Neil Shen’s SJTU Medical Research Fund. **Zheng J. Ma** is affiliated with Beijing Li-Heng Medical Technologies, Ltd, which designed the ICG device used in this study. This affiliation did not play a role in the study design, data collection and analysis, decision to publish, or preparation of the manuscript. In the past three years, **Harlan Krumholz** received expenses and/or personal fees from UnitedHealth, Element Science, Aetna, Reality Labs, Tesseract/4Catalyst, F-Prime, the Siegfried and Jensen Law Firm, Arnold and Porter Law Firm, and Martin/Baughman Law Firm. He is a co-founder of Refactor Health and HugoHealth, and is associated with contracts, through Yale New Haven Hospital, from the Centers for Medicare & Medicaid Services and through Yale University from Johnson & Johnson. Such competing interests do not alter our adherence to PLOS ONE policies on sharing data and materials. The other co-authors report no potential competing interests.

**4. We note that you have indicated that data from this study are available upon request. PLOS only allows data to be available upon request if there are legal or ethical restrictions on sharing data publicly. For more information on unacceptable data access restrictions, please see** [**http://journals.plos.org/plosone/s/data-availability#loc-unacceptable-data-access-restrictions**](http://journals.plos.org/plosone/s/data-availability#loc-unacceptable-data-access-restrictions)**.**

**In your revised cover letter, please address the following prompts:**

**a) If there are ethical or legal restrictions on sharing a de-identified data set, please explain them in detail (e.g., data contain potentially sensitive information, data are owned by a third-party organization, etc.) and who has imposed them (e.g., an ethics committee). Please also provide contact information for a data access committee, ethics committee, or other institutional body to which data requests may be sent.**

**b) If there are no restrictions, please upload the minimal anonymized data set necessary to replicate your study findings as either Supporting Information files or to a stable, public repository and provide us with the relevant URLs, DOIs, or accession numbers. For a list of acceptable repositories, please see** [**http://journals.plos.org/plosone/s/data-availability#loc-recommended-repositories**](http://journals.plos.org/plosone/s/data-availability#loc-recommended-repositories)**.**

**We will update your Data Availability statement on your behalf to reflect the information you provide.**

Response: We updated the data availability statement as shown below. To clarify, the data used in this study was provided by iKang Health Group to be analyzed by to SJTU-Yale Joint Center for Biostatistics in Shanghai, China. Although deidentified, data are from a third party and we are restricted to share it publicly. Data requests, however, can be submitted to iKang Health Group for consideration, and we made the code used to analyze the data publicly available (<https://www.doi.org/10.5281/zenodo.5931975>).

Methods, page 3, lines 51-56 (underlined text is new):

**Data source**

iKang Health Group provided the de-identified data used in this study to SJTU-Yale Joint Center for Biostatistics in Shanghai, China. This is where all data are stored and all analyses were performed. Requests for data can be submitted to the iKang Health Group at [maozhen.zhang@ikang.com](mailto:maozhen.zhang@ikang.com). The code used to analyze the data is publicly available at <https://www.doi.org/10.5281/zenodo.5931975>.

**5. Please note that in order to use the direct billing option the corresponding author must be affiliated with the chosen institute. Please either amend your manuscript to change the affiliation or corresponding author, or email us at** [**plosone@plos.org**](mailto:plosone@plos.org) **with a request to remove this option.**

Response: We confirm that the corresponding author, Harlan M. Krumholz, is affiliated with Yale University with whom PLOS ONE has the direct billing option. We edited his affiliations in the title page.

**6. Thank you for stating the following in the Financial Disclosure section: "This study was self-funded."**

**We note that one or more of the authors are employed by a commercial company: "iKang Healthcare Group, Inc., Shanghai, China and Beijing Li-Heng Medical Technologies, Ltd, Beijing, China.**

**a. Please provide an amended Funding Statement declaring this commercial affiliation, as well as a statement regarding the Role of Funders in your study. If the funding organization did not play a role in the study design, data collection and analysis, decision to publish, or preparation of the manuscript and only provided financial support in the form of authors' salaries and/or research materials, please review your statements relating to the author contributions, and ensure you have specifically and accurately indicated the role(s) that these authors had in your study. You can update author roles in the Author Contributions section of the online submission form.**

**Please also include the following statement within your amended Funding Statement. 
“The funder provided support in the form of salaries for authors [insert relevant initials], but did not have any additional role in the study design, data collection and analysis, decision to publish, or preparation of the manuscript. The specific roles of these authors are articulated in the ‘author contributions’ section.”
If your commercial affiliation did play a role in your study, please state and explain this role within your updated Funding Statement.**

**b. Please also provide an updated Competing Interests Statement declaring this commercial affiliation along with any other relevant declarations relating to employment, consultancy, patents, products in development, or marketed products, etc.**

**Within your Competing Interests Statement, please confirm that this commercial affiliation does not alter your adherence to all PLOS ONE policies on sharing data and materials by including the following statement: "This does not alter our adherence to  PLOS ONE policies on sharing data and materials.” (as detailed online in our guide for authors** [**http://journals.plos.org/plosone/s/competing-interests**](http://journals.plos.org/plosone/s/competing-interests)**) . If this adherence statement is not accurate and  there are restrictions on sharing of data and/or materials, please state these. Please note that we cannot proceed with consideration of your article until this information has been declared.**

**Please include both an updated Funding Statement and Competing Interests Statement in your cover letter. We will change the online submission form on your behalf.**

Response: We confirm that such affiliation did not have any role in the study design, data collection, data analysis, manuscript preparation, or decision to publish, nor was any author compensated for this project in any way.

The revised and updated disclosures are detailed in Comment 3 above.

**7. Please include your full ethics statement in the ‘Methods’ section of your manuscript file. In your statement, please include the full name of the IRB or ethics committee who approved or waived your study, as well as whether or not you obtained informed written or verbal consent. If consent was waived for your study, please include this information in your statement as well.**

Response: We have updated our ethics statement, as shown below.

Methods section, pages 6 and 7, lines 132-136 (underlined text is new):

**Ethics statement**

This project received an exemption for review from the Institutional Review Board at Yale School of Medicine and at Shanghai Jiao Tong University College of Biotechnology as we used de-identified data provided by the iKang Health group. Given that the de-identified data were provided by a third party, we did not need to collect consent for analysis.

**REVIEWER 1**

**Comment 1: It should be clear in the title and abstract that the topic is about non invasive hemodynamic data assessment.**

Response: Thank you for your suggestion. We modified the study title, as shown below (underlined text is new)

**Title:** Hemodynamic Differences Between Women and Men with Elevated Blood Pressure in China: A Non-Invasive Assessment of 45,082 Adults Using Impedance Cardiography

**Comment 2: A flow chart is needed.**

Response: Thank you for your suggestion. We moved the study population flowchart from the supporting information document to the main text as new Figure 1 (shown below):

**Figure 1**. Study population flowchart

Legend: Abbreviations: ICG, impedance cardiography; DBP, diastolic blood pressure; SBP, systolic blood pressure; SV, stroke volume; HR, heart rate; Z0, baseline impedance.

**Comment 3: The authors should give strong reason of why there is a restriction on the availability of data.**

Response: The data used in this study was provided by iKang Health Group to be analyzed by the SJTU-Yale Joint Center for Biostatistics in Shanghai, China. Although deidentified, data are from a third party and we are restricted from sharing it publicly. Data requests, however, can be submitted to iKang Health Group for consideration, and we made the code used to analyze the data publicly available (<https://www.doi.org/10.5281/zenodo.5931975>). We updated our data availability statement to reflect this, as below.

Methods, page 3, lines 51-56 (underlined text is new):

Data source

iKang Health Group provided the de-identified data used in this study to SJTU-Yale Joint Center for Biostatistics in Shanghai, China. This is where all data are stored and all analyses were performed. Requests for data can be submitted to the iKang Health Group at [maozhen.zhang@ikang.com](mailto:maozhen.zhang@ikang.com). The code used to analyze the data is publicly available at <https://www.doi.org/10.5281/zenodo.5931975>.

**Comment 4: In the methodology, the diagnosis of elevated blood pressure relies only on single blood pressure measurement. This is the major concern of the current analysis. Single measurement by automated device is not enough to classify patients regarding their blood pressure level. This point should be properly discussed and defended.**

Response: We recognize that using a single measurement is not ideal because of the possible measurement-to-measurement variability in a single individual. It is possible that we identified individuals as hypertensive based on the single measurement that would not have been included if an average of 2 or more measurements were used instead. However, we also performed a sensitivity analysis using higher blood pressure cutoffs (SBP≥140 mmHg, DBP≥90 mmHg), in which likelihood of inadequately identifying hypertensive individuals is decreased. The findings of this sensitivity analysis were consistent with those of the main analysis.

Furthermore, in all participating sites, blood pressure was measured following a standardized protocol. Although a single measurement may compromise the precision of an individual measurement, the impact of this limitation is reduced by the fact that all sites followed the same protocol with the same calibrated device, regardless of the participants’ sex. We have expanded the limitations, as shown below:

Discussion section, page 17, lines 340-344 (underlined text is new):

Third, a single BP measurement was recorded per participant, which could have affected the precision in this variable. However, all study centers followed the same standardized protocol for men and women, as described in the Methods section, to reduce inter-observer variability. Moreover, such a compromise in precision would most likely shift the observed sex differences towards the null rather than towards significance.

**Comment 5: The authors should justify the use of ICG, the authors should mention the guidelines recommendations or FDA or international medical institutes that approve the use of ICG for such purposes.**

Response: The non-invasive nature of ICG measurement makes it an ideal technology for monitoring hemodynamic parameters in clinics. We leveraged data in clinics where they were being routinely used.

Although there are no guideline recommendations for routine hemodynamic measurement using impedance cardiography, many devices that use ICG have been approved by the FDA for such purposes in different scenarios. Below are some of the FDA 510(k) premarket notifications that support the use of devices using ICG for hemodynamic assessment:

- Approval K080941: intended to use ICG among patients with cardiovascular disease who need cardiac assessment. Available from:
  - <https://www.accessdata.fda.gov/scripts/cdrh/cfdocs/cfpmn/pmn.cfm?ID=K080941>
- Approval K172196: intended to use ICG among patients who are expected to be intubated for less than 24 hours. Available from:
  - <https://www.accessdata.fda.gov/scripts/cdrh/cfdocs/cfpmn/pmn.cfm?ID=K172196>
- Approval K160899: intended to use ICG among patients with fluid-management disorders such as heart failure, chronic kidney disease, or who use diuretics. Available from:
  - <https://www.accessdata.fda.gov/scripts/cdrh/cfdocs/cfpmn/pmn.cfm?ID=K160899>
- Approval K041434: intended to use ICG among patients in hospital areas, including clinics. Available from:
  - <https://www.accessdata.fda.gov/scripts/cdrh/cfdocs/cfpmn/pmn.cfm?ID=K041434>
- Approval K110645: intended to use ICG for general hemodynamic monitoring. Available from:
  - <https://www.accessdata.fda.gov/scripts/cdrh/cfdocs/cfpmn/pmn.cfm?ID=K110645>

The device used in our study has been validated against invasive thermodilution and echocardiography in different scenarios, as mentioned in the methods section (see below).

Methods, page 5, lines 90-94:

The ICG device used (CHM T3002/P3005, designed by Beijing Li-Heng Medical Technologies, Ltd, manufactured by Shandong Baolihao Medical Appliances, Ltd.) was developed based on improved hardware and advanced digital filtering algorithms,[1] and has been validated versus both invasive thermodilution and non-invasive echocardiography in different settings.[2-4]

We defer to the editors whether to cite in the main text the FDA 510(k) documents listed above.

References:

1.	Ma L. Development and application of the latest model of the cardiac hemodynamics monitoring system. International J Cardiovasc Med. 2009;10:11.

2.	An X-g, Zhao Y, Gao J. Clinic evaluation of noninvasive hemodynamic monitoring in patients undergoing coronary artery surgery. Chin J Cardiovasc Rev. 2008;2:010.

3.	Hong H, Jin X, Pan C, Gao X, Liu M, Jiang H, Ge J. Analysis of the correlation between non-invasive hemodynamic monitor and cardiac echocardiography on the evaluation of cardiac function. Chinese Journal of Medical Instrumentation. 2009;33:328-331.

4.	Chen W, Huang D, Zeng J, Deng R, Deng G, Chen W, Zou Y. Application of noninvasive cardiac hemodynamic monitor for ICU critically ill patients. Hainan Medical Journal. 2018;29:15.

**Comment 6: In the results, there was significant difference in the age of women versus men (i.e., 54 vs 45, p <0.001). This significant difference still presents after subgroups analysis of <50 yr or > 50 yr (i.e., p<0.001). This important point should be defended and discuss as significant differences in age would be important confounder and obtaining reliable results needs proper adjustment of the analysis in regard to age.**

Response: Indeed, there were important differences in the mean age between women and men that could influence our findings. We believe, however, that we robustly accounted for such differences in 2 major ways, listed below:

1. We performed a sequentially adjusted linear regression, shown in Table 2. Age was included in Adjusted Model 1 and Adjusted Model 2, without significantly altering the association of female sex and the hemodynamic indicators (either overall or stratified at age 50). In these multivariable analyses, we found that women <50 years old had lower systemic vascular resistance index and higher cardiac index than men of their same age group, whereas among those ≥50 years old, women had higher systemic vascular resistance index but lower cardiac index. Please find Table 2 at the end of this document.
2. We also performed a sensitivity analysis using a nearest neighbor propensity score matching, using region, age, SBP, DBP, and BMI. In this sensitivity analysis, showed in S2 Table (also at the end of this document), findings in magnitude and direction of the multivariable linear regression were similar, with the exception of no significant difference in systemic vascular resistance index among those <50 years old.

Thus, the observed sex differences are not likely to arise just from differences in the mean age of our study sample. Nonetheless, it should be clear that although we found statistically significant differences in the mean of the hemodynamic variables studied, the main finding is the heterogeneity in their distribution such that sex was not determinative of the hemodynamic profile, as below:

Discussion section, pages 15-16, lines 299-311 (underlined relevant text):

Our study also has important implications for personalizing the care of patients presenting to an outpatient clinic with elevated blood pressure. As hypertension remains as one of the biggest public health challenges worldwide, there is urgency in determining possible ways of improving its treatment efficacy by using personalized therapies tailored to each patient’s characteristics. Although we and other studies have shown that, on average, there are significant hemodynamic differences by sex, we also found that there is substantial same-sex heterogeneity in the hemodynamic profile. Notably, as hypertension prevalence and arterial stiffening increases among women after menopause, the distribution overlap of CI and SVRI among those older than 50 years was almost the same between women and men. Thus, our study indicates that sex alone is not a good proxy of the underlying hemodynamic patterns of patients with elevated blood pressure and should not be used clinically to assume the hypertension phenotype. Instead, it is necessary to measure these parameters directly if information about the hemodynamic profile is considered necessary to guide the antihypertensive therapy.

Nonetheless, we added this as a limitation of our analysis, as shown below:

Discussion section, page 17, lines 345-348 (underlined text is new):

Fourth, although we performed a robust main analysis, which included adjustment for multiple confounders, and a sensitivity analysis that used propensity score matching, we cannot rule out that the differences observed between men and women were due to residual confounding.

**Comment 7: The current manuscript does not have data regarding long term follow up to see if the current hemodynamic differences have clinical impact on long term prognosis or not. This point should be properly defended.**

Response: Although we lack such information, we agree on the importance of investigating the long-term association of different hemodynamic phenotypes. However, such an objective is outside the scope of our study which, cross-sectional in design, aimed to evaluate the differences between men and women in hemodynamic profiles in an outpatient setting.

Discussion section, page 15, lines 289-298 (underlined text is new or edited):

Altogether, such findings might suggest that the underlying mechanisms of elevated blood pressure might differ by sex, particularly among young individuals. The clinical implications of these sex differences are limited because of the high same-sex heterogeneity in these parameters that we observed. Beyond its potential implications for treatment adjustment or initiation, understanding if these differences in hypertension phenotypes are implicated in the known sex differences in terms of risk of subsequent cardiovascular outcomes is still uncertain and deserves further investigation. Furthermore, there is a need for longitudinal studies that help understand how chronic exposure to different hypertension phenotypes is associated with clinical outcomes, and if there are sex differences in such associations.

We also edited our limitations to better express this, as shown below.

Discussion, page 16, lines 314-318 (underlined text is new):

The results from our study should be interpreted in light of the following limitations. First, our findings only represent a snapshot of individuals’ hemodynamic status, preventing us from assessing longitudinal clinical outcomes and pathological adaptations that may occur with long-term exposure to a particular hypertension phenotype, including structural and physiological changes in heart and vessels.

**Comment 8: The clinical implications of the current study should be clear and practical.**

**Regards**

Response: Thank you. We edited the clinical implications paragraph to made it clearer, as below.

Discussion section, pages 15 and 16, lines 299-311 (underlined text is new or edited):

Our study also has important implications for personalizing the care of patients presenting to an outpatient clinic with elevated blood pressure. As hypertension remains as one of the biggest public health challenges worldwide,[1] there is urgency in determining possible ways of improving its treatment efficacy by using personalized therapies tailored to each patient’s characteristics. Although we and other studies have shown that, on average, there are significant hemodynamic differences by sex, we also found that there is substantial same-sex heterogeneity. Notably, as hypertension prevalence and arterial stiffening increase among women after menopause,[2-4] the distribution overlap of CI and SVRI among those older than 50 years was almost complete between women and men. Thus, our study indicates that sex alone is not a good proxy of the underlying hemodynamic patterns of patients with elevated blood pressure and should not be used clinically to assume the hypertension phenotype. Instead, it is necessary to measure these parameters directly if information about the hemodynamic profile is considered necessary to guide the antihypertensive therapy.

References:

1. Global, regional, and national comparative risk assessment of 84 behavioural, environmental and occupational, and metabolic risks or clusters of risks for 195 countries and territories, 1990-2017: a systematic analysis for the Global Burden of Disease Study 2017. Lancet (London, England). 2018;392(10159):1923-94.

2. Cheng S, Xanthakis V, Sullivan LM, Vasan RS. Blood pressure tracking over the adult life course: patterns and correlates in the Framingham heart study. Hypertension. 2012;60(6):1393-9.

3. Zhou Y, Zhou X, Guo X, Sun G, Li Z, Zheng L, et al. Prevalence and risk factors of hypertension among pre- and post-menopausal women: a cross-sectional study in a rural area of northeast China. Maturitas. 2015;80(3):282-7.

4. Lu Y, Pechlaner R, Cai J, Yuan H, Huang Z, Yang G, et al. Trajectories of age-related arterial stiffness in Chinese men and women. Journal of the American College of Cardiology. 2020;75(8):870-80.

**REVIEWER 2**

**Comment 1: The cofounders missing some important factors as presence of diabetes, the types of hypertension ,the risk factors as dyslipidemia and the existence of chronic kidney disease. All these factors can contribute in risk stratification and further management of hypertension.**

Response: Our aim was to describe sex differences in a hemodynamic snapshot of people with elevated blood pressure who visited an outpatient setting, regardless of their current management or chronic conditions. This is a real-world, pragmatic study showing that among a large outpatient clinic, there is marked heterogeneity in profiles and that sex is significantly associated with those profiles—but that there is a lot of overlap of the profiles of men and women.

We added the lack of comorbidities to the study’s limitations, as shown below.

Limitations, page 16, lines 319-324 (underlined text is new):

Second, although we had highly detailed hemodynamic information about each individual, the sociodemographic and clinical information (including comorbidities that may affect hemodynamic status) available for our analyses was limited. Of great importance is the lack of information regarding current antihypertensive medication usage, which could alter the hemodynamic phenotype and inclusion criteria (e.g., beta-blockers lowering CO or a patient with controlled hypertension not being included).

**Comment 2: the age 50 is not clear as not standard for old age as WHO recommendations.**

Response: We used 50 years not because it represents the age at which people are consider elder, but because it is the mean age of menopause among women in China.[1-3] See below

Methods section, page 5, lines 96-103 (relevant text is underlined):

**Variable definitions**

We described demographic characteristics and hemodynamic parameters of blood pressure in women and men overall and by age. Considering that the mean age of natural menopause in China is reported as approximately 50 years of age, we stratified our study population as <50 years old and ≥50 years old. We used the World Health Organization recommended cutoff values for BMI classification in Asian populations, defining underweight as <18.5 kg/m^2^, normal weight from 18.5 kg/m^2^ to <23 kg/m^2^, overweight from 23 kg/m^2^ to <27.5 kg/m^2^, and obesity as ≥27.5 kg/m^2^.

To make it clearer, we mention it in the introduction as shown below.

Introduction section, pages 2 and 3, lines 41-48 (underlined text is new):

Accordingly, we used data from tens of thousands of individuals with elevated blood pressure from an outpatient setting in China to evaluate the overall patterns of sex differences in hemodynamic variables and to determine how these sex hemodynamic differences may vary with age. We also aimed to evaluate the distribution of these variables among women and men, and to what extent they overlap by sex. Furthermore, we stratified our analysis at the mean age of menopause in China because of its potential association with hemodynamic changes. Results from this study can advance our understanding of the association of sex with hemodynamic patterns in people with hypertension and suggest if sex could be used to guide therapy.

References:

1.	Dorjgochoo T, Kallianpur A, Gao YT, Cai H, Yang G, Li H, et al. Dietary and lifestyle predictors of age at natural menopause and reproductive span in the Shanghai Women's Health Study. Menopause. 2008;15(5):924-33.

2.	Wang M, Gong WW, Hu RY, Wang H, Guo Y, Bian Z, et al. Age at natural menopause and associated factors in adult women: Findings from the China Kadoorie Biobank study in Zhejiang rural area. PLoS One. 2018;13(4):e0195658.

3.	Song L, Shen L, Li H, Liu B, Zheng X, Zhang L, et al. Age at natural menopause and hypertension among middle-aged and older Chinese women. J Hypertens. 2018;36(3):594-600.

**Comment 3: in table 1 what is meant by predominantly cardiac**

Response: As stated in the footnote of Table 1 (found at the end of this document) and in the methods section, we defined a predominantly cardiac phenotype as high cardiac index with low or normal systemic vascular resistance index. This definition is consistent with previous research.[1-3]

Methods section, page 5, lines 96-107 (relevant text is underlined):

**Variable definitions**

We described demographic characteristics and hemodynamic parameters of blood pressure in women and men overall and by age. Considering that the mean age of natural menopause in China is reported as approximately 50 years of age, we stratified our study population as <50 years old and ≥50 years old. We used the World Health Organization recommended cutoff values for BMI classification in Asian populations, defining underweight as <18.5 kg/m^2^, normal weight from 18.5 kg/m^2^ to <23 kg/m^2^, overweight from 23 kg/m^2^ to <27.5 kg/m^2^, and obesity as ≥27.5 kg/m^2^. We defined a predominantly vascular hypertension phenotype as high SVRI (>2400 dynes·sec·cm^-5^·m^2^) with a low or normal CI (<2.5 L/min/m^2^ or 2.5–4 L/min/m^2^, respectively), and predominantly cardiac hypertension phenotype as high CI (>4 L/min/m^2^) with low or normal SVRI (<2000 dynes·sec·cm^-5^·m^2^ or 2000–2400 dynes·sec·cm^-5^·m^2^, respectively).

References:

1. Lu Y, Wang L, Wang H, Gu J, Ma ZJ, Lian Z, et al. Effectiveness of an impedance cardiography guided treatment strategy to improve blood pressure control in a real-world setting: results from a pragmatic clinical trial. Open Heart. 2021;8(2): e001719.
2. Mahajan S, Gu J, Caraballo C, Lu Y, Spatz ES, Zhao H, et al. Relationship of age with the hemodynamic parameters in individuals with elevated blood pressure. Journal of the American Geriatrics Society. 2020;68(7):1520-1528.
3. Mahajan S, Gu J, Lu Y, Khera R, Spatz ES, Zhang M, et al. Hemodynamic phenotypes of hypertension based on cardiac output and systemic vascular resistance. The American Journal of Medicine. 2020;133(4):e127-e139.

Additional comment from the authors: please note that, for conciseness and consistency in the data reporting across text and tables, we edited the P values to express P values to 2 digits to the right of the decimal point, or to 3 digits if <0.01. During our thorough revision we also detected that a few P values in Table 1, Table S1, and Table S2 needed to be updated because of initial entry error. We apologize for the initial typographical errors, which have been corrected and tracked, and we confirm that none of the changes affect our findings or conclusions.

**Tables**

**Table 1. Sex differences in clinical and hemodynamic variables by age group among adults with elevated blood pressure.**

|  | **All** | | | **< 50 years old** | | | **≥ 50 years old** | | |
| --- | --- | --- | --- | --- | --- | --- | --- | --- | --- |
|  | Women  N=15,888 | Men  N= 29194 | P value | Women  N = 4,384 | Men  N = 15,512 | P value | Women  N = 11,504 | Men  N = 13,682 | P value |
| Age (years) | 54.5 (11.8) | 48.0 (13.0) | <0.001 | 39.3 (8.1) | 38.0 (7.3) | <0.001 | 60.2 (6.9) | 59.5 (7.2) | <0.001 |
| BMI (kg/m2) | 24.4 (3.5) | 25.5 (3.2) | <0.001 | 23.5 (3.7) | 25.7 (3.4) | <0.001 | 24.8 (3.3) | 25.2 (3.0) | <0.001 |
| Obesity* | 2733 (17.2%) | 6858 (23.49%) | <0.001 | 585 (13.34%) | 4057 (26.15%) | <0.001 | 2148 (18.67%) | 2801 (20.47%) | <0.001 |
| Region |  |  | <0.001 |  |  | <0.001 |  |  | <0.001 |
| East | 5965 (37.54%) | 13275 (45.47%) |  | 1956 (20.86%) | 7419 (79.14%) |  | 4009 (40.64%) | 5856 (59.36%) |  |
| North | 3665 (23.07%) | 4156 (14.24%) |  | 779 (29.59%) | 1854 (70.41%) |  | 2886 (55.63%) | 2302 (44.37%) |  |
| South | 2737 (17.23%) | 4317 (14.79%) |  | 686 (22.66%) | 2341 (77.34%) |  | 2051 (50.93%) | 1976 (49.07%) |  |
| Southwest | 3521 (22.16%) | 7446 (25.51%) |  | 963 (19.81%) | 3898 (80.19%) |  | 2558 (41.89%) | 3548 (58.11%) |  |
| Blood pressure (mmHg) |  |  |  |  |  |  |  |  |  |
| Systolic | 139.0 (15.7) | 136.7 (13.8) | <0.001 | 131.2 (13.2) | 133.7 (12.2) | <0.001 | 142.0 (15.6) | 140.1 (14.8) | <0.001 |
| Diastolic | 82.6 (9.00) | 85.6 (8.9) | <0.001 | 83.3 (7.9) | 85.3 (8.9) | <0.001 | 82.4 (9.4) | 86.01 (9.0) | <0.001 |
| Hypertension phenotype |  |  |  |  |  |  |  |  |  |
| Predominantly cardiac**^†^** | 2559 (16.11%) | 5185 (17.76%) | <0.001 | 1382 (31.52%) | 3495 (22.53%) | <0.001 | 1177 (10.23%) | 1690 (12.35%) | <0.001 |
| Predominantly vascular**^‡^** | 9780 (61.56%) | 16396 (56.16%) | <0.001 | 1666 (38.00%) | 7197 (46.40%) | <0.001 | 8114 (70.53%) | 9199 (67.23%) | <0.001 |
| Low/normal CI & low/normal SVRI | 3531 (22.22)% | 7548 (25.86%) | <0.001 | 1,330 (30.34%) | 4,790(30.88%) | 0.50 | 2,201 (19.13%) | 2,758 (20.16%) | 0.04 |
| High CI & high SVRI | 18 (0.11%) | 65 (0.22%) | 0.01 | 6 (0.14%) | 30 (0.19%) | 0.56 | 12 (0.10%) | 35 (0.26%) | 0.01 |
| ICG parameters |  |  |  |  |  |  |  |  |  |
| Heart rate (bpm) | 69.4 (11.4) | 69.5 (11.3) | 0.63 | 72.6 (11.9) | 70.8 (11.1) | <0.001 | 68.2 (10.9) | 68.0 (11.3) | 0.15 |
| Stroke volume (mL) | 72.9 (18.6) | 88.8 (21.5) | <0.001 | 80.0 (18.8) | 93.0 (21.6) | <0.001 | 70.2 (17.7) | 84.0 (20.5) | <0.001 |
| CO (L/min) | 5.0 (1.4) | 6.1 (1.5) | <0.001 | 5.8 (1.4) | 6.5 (1.4) | <0.001 | 4.7 (1.2) | 5.6 (1.4) | <0.001 |
| CI (L/min/m^2^) | 3.2 (0.8) | 3.3 (0.8) | <0.001 | 3.6 (0.9) | 3.5 (0.7) | <0.001 | 3.0 (0.8) | 3.2 (0.7) | <0.001 |
| SVR (dynes·sec·cm^-5^) | 1744 (523) | 1433 (389) | <0.001 | 1471 (411) | 1315 (324) | <0.001 | 1848 (524) | 1565 (412) | <0.001 |
| SVRI (dynes·sec·cm^-5^·m^2^) | 2734.1 (809.9) | 2596.3 (677.2) | <0.001 | 2326.0 (658.0) | 2435.1 (598.6) | <0.001 | 2889.6 (808.3) | 2779.2 (713.8) | <0.001 |
| Data are presented as mean (SD) for continuous variables and n (%) for categorical variables.  * Obesity was defined as BMI ≥27.5 kg/m2  † A predominantly cardiac hypertension phenotype was determined by high CI with low or normal SVRI  ‡ Predominantly vascular hypertension phenotype was determined by low or normal CI with high SVRI  Abbreviations: SD= Standard Deviation, BMI= Body Mass Index, ICG= Impedance Cardiography, SVR= Systemic Vascular Resistance, SVRI= Systemic Vascular Resistance Index, CO= Cardiac Output, CI= Cardiac Index. | | | | | | | | | |

**Table 2. Unadjusted and Sequentially-Adjusted Association of Female Sex with Cardiac Output, Cardiac Index, Systemic Vascular Resistance, and Systemic Vascular Resistance Index, Overall and by Age Categories.**

| **Hemodynamic Variable** | **Female Sex β Coefficient (95% CI)** | | |
| --- | --- | --- | --- |
|  | Unadjusted Model | Adjusted Model 1* | Adjusted Model 2† |
| **Cardiac Output,** (L/min) | | | |
| Overall | -1.07 (-1.1, -1.04) | -0.79 (-0.82, -0.77) | -0.78 (-0.8, -0.75) |
| <50 years old | -0.73 (-0.78, -0.68) | -0.67 (-0.71, -0.62) | -0.59 (-0.64, -0.54) |
| ≥50 years old | -0.90 (-0.93, -0.87) | -0.86 (-0.89, -0.83) | -0.86 (-0.89, -0.83) |
|  |  |  |  |
| **Cardiac Index,** (L/min/m^2^) | | | |
| Overall | -0.15 (-0.16, -0.13) | -0.01 (-0.03, 0)^‡^ | -0.08 (-0.09, -0.06) |
| <50 years old | 0.14 (0.12, 0.17) | 0.19 (0.16, 0.21) | 0.07 (0.04, 0.09) |
| ≥50 years old | -0.14 (-0.16, -0.12) | -0.12 (-0.14, -0.1) | -0.15 (-0.16, -0.13) |
|  |  |  |  |
| **Systemic Vascular Resistance,** (dynes·sec·cm^-5^) | | | |
| Overall | 312 (303, 320) | 225 (216, 233) | 230 (221, 238) |
| <50 years old | 156 (144, 167) | 138 (127, 149) | 140 (128, 151) |
| ≥50 years old | 282 (271, 294) | 271 (260, 282) | 274 (262, 285) |
|  |  |  |  |
| **Systemic Vascular Resistance Index,** (dynes·sec·cm^-5^·m^2^) | | | |
| Overall | 137.8 (123.7, 151.8) | 6.0 (-7.6, 19.7)^§^ | 73.5 (60.2, 86.8) |
| <50 years old | -109.1 (-129.7, -88.6) | -146.9 (-166.6, -127.3) | -31.7 (-51.2, -12.2) |
| ≥50 years old | 110.5 (91.7, 129.3) | 88.5 (69.9, 107.2) | 120.4 (102.4, 138.5) |
| * Model 1 was adjusted for age and region.  † Model 2 was adjusted for age, region, and body mass index.  ^‡^P value=0.16  ^§^P value=0.39  All other P values <0.001 | | | |

**S2 Table.** Sex Differences in Clinical and Hemodynamic Variables by Nearest Neighbor Propensity Score Matched Subgroups.

|  | All  1:1 Matching | | | <50 years old  1:1 Matching | | | ≥50 years old  1:1 Matching | | |
| --- | --- | --- | --- | --- | --- | --- | --- | --- | --- |
|  | Women  N=15,888 | Men  N= 15,888 | P value | Women  N = 4,384 | Men  N = 4,384 | P value | Women  N = 11,504 | Men  N = 11,504 | P value |
| Age, years mean (SD) | 54.46 (11.83) | 53.45 (12.72) | <0.001 | 39.3 (8.07) | 39.27 (7.5) | 0.84 | 60.24 (6.9) | 59.68 (7.24) | <0.001 |
| BMI (kg/m2), mean (SD) | 24.4 (3.49) | 24.53 (2.95) | <0.001 | 23.49 (3.72) | 23.65 (3.3) | 0.04 | 24.75 (3.33) | 24.97 (2.94) | <0.001 |
| Obese (BMI ≥27.5 kg/m2), n(%) | 2733 (17.2%) | 2284 (14.38%) | <0.001 | 585 (13.34%) | 483 (11.02%) | <0.001 | 2148 (18.67%) | 2086 (18.13%) | 0.30 |
| Region, n (%) |  |  | <0.001 |  |  | 0.26 |  |  | <0.001 |
| East | 5965 (48.74%) | 6273 (51.26%) |  | 1956 (50.46%) | 1920 (49.54%) |  | 4009 (46.58%) | 4597 (53.42%) |  |
| North | 3665 (51.84%) | 3405 (48.16%) |  | 779 (47.94%) | 846 (52.06%) |  | 2886 (55.98%) | 2269 (44.02%) |  |
| South | 2737 (50.39%) | 2695 (49.61%) |  | 686 (49.67%) | 695 (50.33%) |  | 2051 (52.32%) | 1869 (47.68%) |  |
| South West | 3521 (50.04%) | 3515 (49.96%) |  | 963 (51.06%) | 923 (48.94%) |  | 2558 (48.02%) | 2769 (51.98%) |  |
| Blood pressure in mmHg, mean (SD) |  |  |  |  |  |  |  |  |  |
| Systolic | 139.02 (15.71) | 138 (13.98) | <0.001 | 131.21 (13.19) | 131.52 (11.06) | 0.04 | 142 (15.57) | 140.36 (14.57) | <0.001 |
| Diastolic | 82.64 (9.00) | 83.62 (8.39) | <0.001 | 83.32 (7.85) | 83.37 (8.05) | 0.23 | 82.37 (9.38) | 84.61 (8.32) | <0.001 |
| ICG parameters, mean (SD) |  |  |  |  |  |  |  |  |  |
| CO (L/min) | 5.01 (1.35) | 5.87 (1.43) | <0.001 | 5.75 (1.41) | 6.39 (1.42) | <0.001 | 4.73 (1.21) | 5.63 (1.35) | <0.001 |
| CI (L/min/m2) | 3.19 (0.84) | 3.31 (0.78) | <0.001 | 3.64 (0.88) | 3.59 (0.77) | 0.013 | 3.02 (0.76) | 3.18 (0.74) | <0.001 |
| SVR  (dynes·sec·cm-5) | 1743.94 (523.42) | 1475.6 (406.58) | <0.001 | 1470.91 (411.34) | 1306.85 (315.19) | <0.001 | 1847.99 (524.09) | 1552.76 (410.64) | <0.001 |
| SVRI  (dynes·sec·cm-5·m2) | 2734.1 (809.92) | 2606.44 (691.7) | <0.001 | 2325.95 (658.03) | 2322.25 (556.02) | 0.78 | 2889.64 (808.27) | 2741.28 (702.93) | <0.001 |
| SD= Standard Deviation, BMI= Body Mass Index, ICG= Impedance Cardiography, SVR= Systemic Vascular Resistance, SVRI= Systemic Vascular Resistance Index, CO= Cardiac Output, CI= Cardiac Index.  Propensity Score Generation Model: Gender ~ Age + BMI + SBP + DBP + Region | | | | | | | | | |
