# Supplementary material for: Hemodynamic differences between women and men with elevated blood pressure in China: A non-invasive assessment of 45,082 adults using impedance cardiography
Source: PLoS One. 2022 Jun 14;17(6):e0269777. doi: 10.1371/journal.pone.0269777 (PMC9197037; doi:10.1371/journal.pone.0269777)
Supplement: Attachment — Submitted filename: PONE-D-21-16564_R2_Response_2022_05_12.docx [file pone.0269777.s008.docx]

**Response To Comments**

PONE-D-21-16564: Hemodynamic Differences Between Women and Men with Elevated Blood Pressure in China: A non-invasive assessment of 45,082 adults using impedance cardiography

We have responded to each comment (reproduced in **bold**) and detailed our changes to the manuscript. In this document, quoted text is presented indented and new additions (or relevant text when specified) underlined. The page and line numbers that we reference below are based on the clean version of our revised manuscript. New references are listed at the end of each response.

**ACADEMIC EDITOR**

**Comment 1: Thank you for submitting your manuscript to PLOS ONE. After careful consideration, we feel that it has merit but does not fully meet PLOS ONE’s publication criteria as it currently stands. Therefore, we invite you to submit a revised version of the manuscript that addresses the points raised during the review process.**

**The authors improved the manuscript but appear to have ignored comments of reviewer 2. If they respond sufficiently to these comments the manuscript can be accepted. Please check carefully once more the initial comments of reviewer 2 as listed below:**

**Response:** We appreciate the academic editor comments and the opportunity to revise our work. This may be a misunderstanding. We did respond to the Reviewer 2 in our initial response letter and made some modifications to the manuscript accordingly. We apologize if our responses were somehow left out from the document that you reviewed. We reviewed each comment once more and included our responses below. Please note that we highlighted these changes in the attached tracked version of the manuscript.

**Comment 2:**

**- The cofounders missing some important factors as presence of diabetes, the types of hypertension ,the risk factors as dyslipidemia and the existence of chronic kidney disease. All these factors can contribute in risk stratification and further management of hypertension.**

**Response**: Our aim was to describe sex differences in a hemodynamic snapshot of people with elevated blood pressure who visited an outpatient setting, regardless of their current management or chronic conditions. This is a real-world, pragmatic study showing that among a large outpatient clinic, there is marked heterogeneity in profiles and that sex is significantly associated with those profiles—but that there is a lot of overlap of the profiles of men and women.

We added the lack of comorbidities to the study’s limitations, as shown below.

Limitations, page 16, lines 321-326 (underlined text is new):

Second, although we had highly detailed hemodynamic information about each individual, the sociodemographic and clinical information (including comorbidities that may affect hemodynamic status) available for our analyses was limited. Of great importance is the lack of information regarding current antihypertensive medication usage, which could alter the hemodynamic phenotype and inclusion criteria (e.g., beta-blockers lowering CO or a patient with controlled hypertension not being included).

**- Comment 3: the age 50 is not clear as not standard for old age as WHO recommendations.**

**Response**: We used 50 years not because it represents the age at which people are consider elder, but because it is the mean age of menopause among women in China.[1-3] See below

Methods section, page 5, lines 98-105 (relevant text is underlined):

**Variable definitions**

We described demographic characteristics and hemodynamic parameters of blood pressure in women and men overall and by age. Considering that the mean age of natural menopause in China is reported as approximately 50 years of age, we stratified our study population as <50 years old and ≥50 years old. We used the World Health Organization recommended cutoff values for BMI classification in Asian populations, defining underweight as <18.5 kg/m^2^, normal weight from 18.5 kg/m^2^ to <23 kg/m^2^, overweight from 23 kg/m^2^ to <27.5 kg/m^2^, and obesity as ≥27.5 kg/m^2^.

To make it clearer, we mention it in the introduction as shown below.

Introduction section, pages 2 and 3, lines 41-48 (underlined text is new):

Accordingly, we used data from tens of thousands of individuals with elevated blood pressure from an outpatient setting in China to evaluate the overall patterns of sex differences in hemodynamic variables and to determine how these sex hemodynamic differences may vary with age. We also aimed to evaluate the distribution of these variables among women and men, and to what extent they overlap by sex. Furthermore, we stratified our analysis at the mean age of menopause in China because of its potential association with hemodynamic changes. Results from this study can advance our understanding of the association of sex with hemodynamic patterns in people with hypertension and suggest if sex could be used to guide therapy.

References:

1.	Dorjgochoo T, Kallianpur A, Gao YT, Cai H, Yang G, Li H, et al. Dietary and lifestyle predictors of age at natural menopause and reproductive span in the Shanghai Women's Health Study. Menopause. 2008;15(5):924-33.

2.	Wang M, Gong WW, Hu RY, Wang H, Guo Y, Bian Z, et al. Age at natural menopause and associated factors in adult women: Findings from the China Kadoorie Biobank study in Zhejiang rural area. PLoS One. 2018;13(4):e0195658.

3.	Song L, Shen L, Li H, Liu B, Zheng X, Zhang L, et al. Age at natural menopause and hypertension among middle-aged and older Chinese women. J Hypertens. 2018;36(3):594-600.

**- Comment 4: in table 1 what is meant by predominantly cardiac**

Response: As stated in the footnote of Table 1 (see underlined footnote in Table 1 below) and in the methods section, we defined a predominantly cardiac phenotype as high cardiac index with low or normal systemic vascular resistance index. This definition is consistent with previous research.[1-3]

Methods section, page 5, lines 98-109 (relevant text is underlined):

**Variable definitions**

We described demographic characteristics and hemodynamic parameters of blood pressure in women and men overall and by age. Considering that the mean age of natural menopause in China is reported as approximately 50 years of age, we stratified our study population as <50 years old and ≥50 years old. We used the World Health Organization recommended cutoff values for BMI classification in Asian populations, defining underweight as <18.5 kg/m^2^, normal weight from 18.5 kg/m^2^ to <23 kg/m^2^, overweight from 23 kg/m^2^ to <27.5 kg/m^2^, and obesity as ≥27.5 kg/m^2^. We defined a predominantly vascular hypertension phenotype as high SVRI (>2400 dynes·sec·cm^-5^·m^2^) with a low or normal CI (<2.5 L/min/m^2^ or 2.5–4 L/min/m^2^, respectively), and predominantly cardiac hypertension phenotype as high CI (>4 L/min/m^2^) with low or normal SVRI (<2000 dynes·sec·cm^-5^·m^2^ or 2000–2400 dynes·sec·cm^-5^·m^2^, respectively).

References:

1. Lu Y, Wang L, Wang H, Gu J, Ma ZJ, Lian Z, et al. Effectiveness of an impedance cardiography guided treatment strategy to improve blood pressure control in a real-world setting: results from a pragmatic clinical trial. Open Heart. 2021;8(2): e001719.
2. Mahajan S, Gu J, Caraballo C, Lu Y, Spatz ES, Zhao H, et al. Relationship of age with the hemodynamic parameters in individuals with elevated blood pressure. Journal of the American Geriatrics Society. 2020;68(7):1520-1528.
3. Mahajan S, Gu J, Lu Y, Khera R, Spatz ES, Zhang M, et al. Hemodynamic phenotypes of hypertension based on cardiac output and systemic vascular resistance. The American Journal of Medicine. 2020;133(4):e127-e139.

**Table 1. Sex differences in clinical and hemodynamic variables by age group among adults with elevated blood pressure.**

|  | **All** | | | **< 50 years old** | | | **≥ 50 years old** | | |
| --- | --- | --- | --- | --- | --- | --- | --- | --- | --- |
|  | Women  N=15,888 | Men  N= 29194 | P value | Women  N = 4,384 | Men  N = 15,512 | P value | Women  N = 11,504 | Men  N = 13,682 | P value |
| Age (years) | 54.5 (11.8) | 48.0 (13.0) | <0.001 | 39.3 (8.1) | 38.0 (7.3) | <0.001 | 60.2 (6.9) | 59.5 (7.2) | <0.001 |
| BMI (kg/m2) | 24.4 (3.5) | 25.5 (3.2) | <0.001 | 23.5 (3.7) | 25.7 (3.4) | <0.001 | 24.8 (3.3) | 25.2 (3.0) | <0.001 |
| Obesity* | 2733 (17.2%) | 6858 (23.49%) | <0.001 | 585 (13.34%) | 4057 (26.15%) | <0.001 | 2148 (18.67%) | 2801 (20.47%) | <0.001 |
| Region |  |  | <0.001 |  |  | <0.001 |  |  | <0.001 |
| East | 5965 (37.54%) | 13275 (45.47%) |  | 1956 (20.86%) | 7419 (79.14%) |  | 4009 (40.64%) | 5856 (59.36%) |  |
| North | 3665 (23.07%) | 4156 (14.24%) |  | 779 (29.59%) | 1854 (70.41%) |  | 2886 (55.63%) | 2302 (44.37%) |  |
| South | 2737 (17.23%) | 4317 (14.79%) |  | 686 (22.66%) | 2341 (77.34%) |  | 2051 (50.93%) | 1976 (49.07%) |  |
| Southwest | 3521 (22.16%) | 7446 (25.51%) |  | 963 (19.81%) | 3898 (80.19%) |  | 2558 (41.89%) | 3548 (58.11%) |  |
| Blood pressure (mmHg) |  |  |  |  |  |  |  |  |  |
| Systolic | 139.0 (15.7) | 136.7 (13.8) | <0.001 | 131.2 (13.2) | 133.7 (12.2) | <0.001 | 142.0 (15.6) | 140.1 (14.8) | <0.001 |
| Diastolic | 82.6 (9.00) | 85.6 (8.9) | <0.001 | 83.3 (7.9) | 85.3 (8.9) | <0.001 | 82.4 (9.4) | 86.01 (9.0) | <0.001 |
| Hypertension phenotype |  |  |  |  |  |  |  |  |  |
| Predominantly cardiac**^†^** | 2559 (16.11%) | 5185 (17.76%) | <0.001 | 1382 (31.52%) | 3495 (22.53%) | <0.001 | 1177 (10.23%) | 1690 (12.35%) | <0.001 |
| Predominantly vascular**^‡^** | 9780 (61.56%) | 16396 (56.16%) | <0.001 | 1666 (38.00%) | 7197 (46.40%) | <0.001 | 8114 (70.53%) | 9199 (67.23%) | <0.001 |
| Low/normal CI & low/normal SVRI | 3531 (22.22)% | 7548 (25.86%) | <0.001 | 1,330 (30.34%) | 4,790(30.88%) | 0.50 | 2,201 (19.13%) | 2,758 (20.16%) | 0.04 |
| High CI & high SVRI | 18 (0.11%) | 65 (0.22%) | 0.01 | 6 (0.14%) | 30 (0.19%) | 0.56 | 12 (0.10%) | 35 (0.26%) | 0.01 |
| ICG parameters |  |  |  |  |  |  |  |  |  |
| Heart rate (bpm) | 69.4 (11.4) | 69.5 (11.3) | 0.63 | 72.6 (11.9) | 70.8 (11.1) | <0.001 | 68.2 (10.9) | 68.0 (11.3) | 0.15 |
| Stroke volume (mL) | 72.9 (18.6) | 88.8 (21.5) | <0.001 | 80.0 (18.8) | 93.0 (21.6) | <0.001 | 70.2 (17.7) | 84.0 (20.5) | <0.001 |
| CO (L/min) | 5.0 (1.4) | 6.1 (1.5) | <0.001 | 5.8 (1.4) | 6.5 (1.4) | <0.001 | 4.7 (1.2) | 5.6 (1.4) | <0.001 |
| CI (L/min/m^2^) | 3.2 (0.8) | 3.3 (0.8) | <0.001 | 3.6 (0.9) | 3.5 (0.7) | <0.001 | 3.0 (0.8) | 3.2 (0.7) | <0.001 |
| SVR (dynes·sec·cm^-5^) | 1744 (523) | 1433 (389) | <0.001 | 1471 (411) | 1315 (324) | <0.001 | 1848 (524) | 1565 (412) | <0.001 |
| SVRI (dynes·sec·cm^-5^·m^2^) | 2734.1 (809.9) | 2596.3 (677.2) | <0.001 | 2326.0 (658.0) | 2435.1 (598.6) | <0.001 | 2889.6 (808.3) | 2779.2 (713.8) | <0.001 |
| Data are presented as mean (SD) for continuous variables and n (%) for categorical variables.  * Obesity was defined as BMI ≥27.5 kg/m2  † A predominantly cardiac hypertension phenotype was determined by high CI with low or normal SVRI  ‡ Predominantly vascular hypertension phenotype was determined by low or normal CI with high SVRI  Abbreviations: SD= Standard Deviation, BMI= Body Mass Index, ICG= Impedance Cardiography, SVR= Systemic Vascular Resistance, SVRI= Systemic Vascular Resistance Index, CO= Cardiac Output, CI= Cardiac Index. | | | | | | | | | |
